# CRISPReader System Sensing the Ets-1 Transcription Factor Can Effectively Identify Cancer Cells

**DOI:** 10.3389/fmolb.2021.672040

**Published:** 2021-05-28

**Authors:** Kang Yang, Yan Zhou, Hongcai Zhong

**Affiliations:** ^1^HuiZhou Municipal Central Hospital, Huizhou, China; ^2^Logistics Management Office, HuiZhou University, Huizhou, China

**Keywords:** Ets-1, CRISPReader, cancer, cancer related signal, gene therapy

## Abstract

By targeting key genes, the CRISPR system can effectively exert its anti-cancer activity. The latest research suggests that the CRISPReader system that regulates gene transcription can effectively target and inhibit bladder cancer cells by sensing transcription factors such as c-Myc and Get-1 in the cell. An interesting question is whether the CRISPReader system can exert its anti-cancer ability against a variety of tumors by sensing the broad-spectrum transcription factor Ets-1. In this work, a CRISPReader system that senses the Ets-1 transcription factor has been constructed. It can effectively identify a variety of cancer cell lines, and specifically induce apoptosis in cancer cells. This study fully confirmed the effectiveness of Ets-1 as a broad-spectrum cancer related signal and provided a new anti-cancer tool based on the CRISPReader system.

## Introduction

CRISPR has become a popular gene editing technology in recent years, which can be used to modify species and investigate gene functions (Lino et al., 2018). The CRISPR nuclease, such as Cas9 protein, can induce DNA double-strand breaks and DNA recombination repair mediated by homologous templates (Doudna and Charpentier, 2014). The mutated dead Cas9, dCas9, can bind to the gene promoter region to regulate gene transcription levels (Gjaltema and Schulz, 2018). By modifying the dCas9 protein or sgRNA, an inducible dCas9 system can be constructed (Dai et al., 2018). The regulation of target genes by dCas protein is activated by sensing exogenous inducers or endogenous proteins within cells (Liu Y. et al., 2016; Liu et al., 2017). This is significant because it successfully realizes the systematic sensation of the key signals of tumor cells. The regulation of downstream gene expression through inducible dCas9 is not only conducive to the specific recognition of cancer cells but also can effectively inhibit the malignant growth of tumors. This provides new strategies for basic research and the clinical treatment of tumors.

A key aspect of the research progress made for the CRISPR transcriptional regulation system is to use the dCas9 transcription activator to bind to its transcription initiation element to replace the promoter to perform gene expression functions. This is a very important technological advancement. The related technology is called “CRISPReader” (Zhan H. et al., 2019), which was originally used to reduce the gene expression cassette of CRISPR so that it can be loaded into AAV vectors with limited capacity. The gene circuit based on CRISPReader can sense transcription factors such as c-Myc and Get-1 in cancer cells (Liu et al., 2020). It can not only effectively identify bladder cancer cells from non-bladder cancer cells, but also effectively inhibit the growth and metastasis of cancer cells. However, this gene circuit is only suitable for the gene therapy of bladder cancer because of the tissue expression specificity of the Get-1 transcription factor.

Ets-1 is a transcription factor that is widely expressed in a variety of tumors (Hahne et al., 2008). Previous studies have found that the TERT promoter of many tumors has high frequency mutations, and the mutated sequence increases the binding site of Ets-1 (Li et al., 2015; Liu L. et al., 2016). TERT is also a broad-spectrum tumor marker, which is conducive to the excessive division of tumor cells.

A more interesting idea is to use CRISPReader to sense Ets-1 and to develop a broad-spectrum anti-cancer tool. In this study, based on the CRISPReader technology, we successfully constructed a gene circuit that senses the endogenous Ets-1 transcription factor in cells. It can identify a variety of cancer cells including bladder cancer and gastric cancer, and can specifically and effectively induce apoptosis of cancer cells.

## Materials and Methods

### Cancer Cell Culture

The cancer cell lines used in this study were purchased from the Institute of Cell Research, Chinese Academy of Sciences (Shanghai, China). Cells were grown in a high-sugar DMEM medium (Qingdao Jieshikang Biotechnology Co., Ltd.) supplemented with 10% fetal bovine serum (Invitrogen) at 37°C in a 5% carbon dioxide atmosphere.

### Construction of the CRISPReader System

The cDNA sequences for sgRNA binding regions were designed, synthesized, and inserted into the corresponding vector, which had been digested with restriction endonucleases. All vectors were transformed into One Shot TOP10 Chemically Competent *E. coli* cells, and the desired expression clones were identified using polymerase chain reaction (PCR) amplification and electrophoresis, and then confirmed with Sanger sequencing.

### Gene Transfection

The above plasmids were transfected into various cancer cells using Lipofectamine 2000 kit according to the operation instructions when the cell density reached 60 ∼70%.

### Luciferase Activity Detection

After transfection for 48 h, cells were obtained. Next, the activities of firefly and ranilla luciferase were analyzed by double luciferase reporting assay (Promega) in line with the manufacturer’s specifications.

#### GFP Reporter Gene Assay

GFP reporter gene assay was also used for studying the regulation of gene expression. A specialized microscope was used to see GFP-expressing cells. The percentage of cells showing green fluorescence was quantified by the Image J software^1^.

#### Cell Apoptosis Assay

Cancer cells were transiently transfected with plasmid vectors, and then a BD flow cytometer (Beijing Delica Biotechnology Co., Ltd.) was used to detect the cells that had been transfected for 48 h and stained by Annexin V and PI in a 6-hole plate, and the experiment was repeated three times.

#### CCK-8 Cell Proliferation Assay

Cancer cells were transiently transfected with plasmid vectors, and then cells were analyzed with CCK-8 assay after being cultured in DMEM medium for 24, 48, and 72 h. For the determination of cell proliferation, a Cell-Counting Kit 8 (Dojindo Laboratories) was used according to the instructions. Fluorescence (450 nm) was recorded using a microplate reader.

### Statistical Analysis

SPSS 21.0 (SPSS, Inc., Chicago, IL, United States) was used to conduct statistical analysis. *T*-test was applied to express the measurement data, which was expressed by the mean number ± standard deviation (*x* ± SD). *P* < 0.05 was considered statistically significant.

## Results

### Construction of a CRISPReader That Senses the Ets-1 Transcription Factor

**FIGURE 1:**
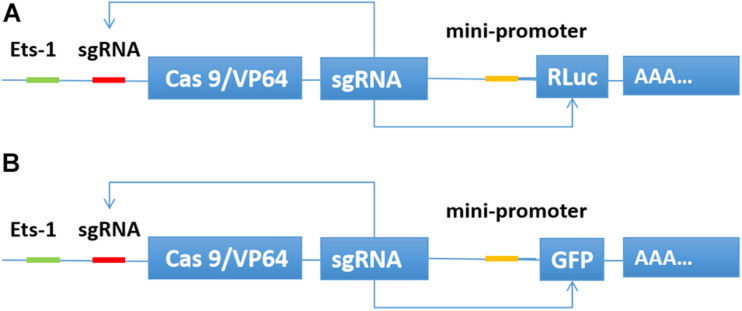
Construction of CRISPReader sensing the Ets-1 transcription factor. When Ets-1 binds to the promoter region, weak expression of Cas9/VP64 can be initiated. After Cas9/VP64 is combined with sgRNA, it can further bind its promoter and drive its own expression. In this way, the expression level of Cas9/VP64 is continuously increased through positive feedback, and then the transcription of luciferase **(A)** or GFP **(B)** is activated.

We inserted the binding sequence of transcription factor Ets-1 into the upstream of the transcription initiation element TATA box. Then, the sgRNA binding site was inserted between the two elements. The Cas9/VP64 and sgRNA expression framework were inserted downstream of the transcription start site. The sgRNA also targets the promoter region of luciferase or GFP. When the expression of the transcription factor Ets-1 in the cell is very low, neither Cas9/VP64 nor sgRNA is expressed. In cancer cells expressing Ets-1, Cas9/VP64 and sgRNA continue to drive their self-expression, and then transcriptionally activate luciferase (Figure 1A) or GFP (Figure 1B).

### Distinguishing Between Bladder Cancer Cells and Normal Cells

**FIGURE 2:**
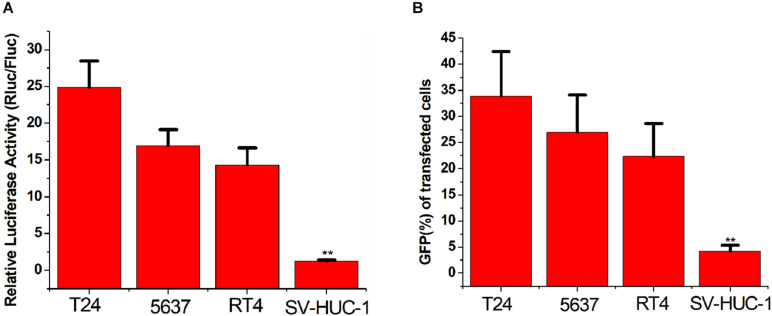
Distinguishing between bladder cancer cells and normal cells. **(A)** Cell luciferase activity was determined using the dual luciferase reporting assay (**p < 0.01). Data were expressed as mean ± SD. **(B)** Cell GFP expression was determined using the GFP reporter assay (**p < 0.01). Data were expressed as mean ± SD.

To verify whether the CRISPReader system can specifically recognize bladder cancer cells by sensing the expression of the transcription factor Ets-1, we transiently transferred the plasmid expressing the system into a series of cancer cells and corresponding normal cells. The luciferase test results suggest that the CRISPReader can significantly activate the expression of luciferase in T24, 5637, RT4 bladder cancer cell lines that highly express Ets-1, but cannot express luciferase in normal urothelial cells SV-HUC-1 (Figure 2A). The results were the same when the reporter gene was GFP (Figure 2B). The difference in the expression of reporter genes suggests that the CRISPReader system can effectively distinguish bladder tumor cells from normal bladder cells.

### Distinguishing Between Gastric Cancer Cells and Normal Cells

**FIGURE 3:**
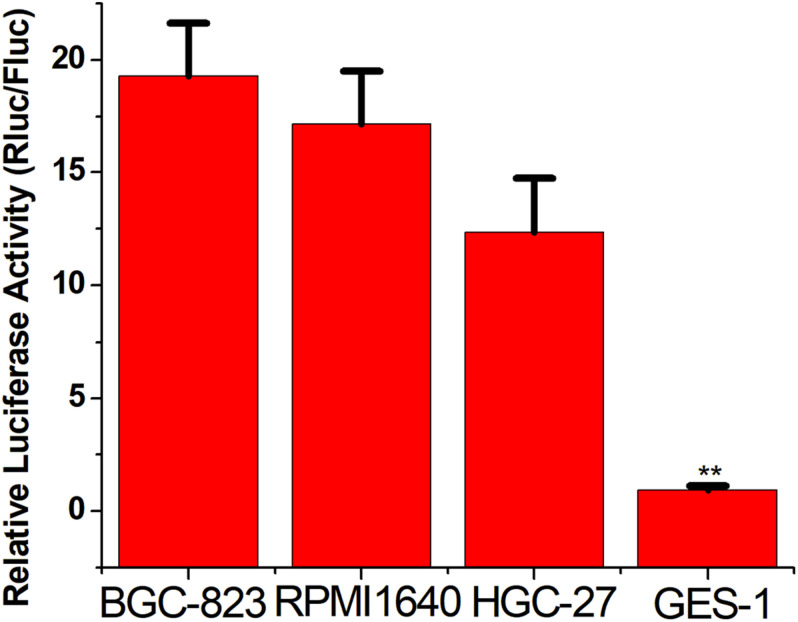
Distinguishing between gastric cancer cells and normal cells. Cell luciferase activity was determined using the dual luciferase reporting assay (**p < 0.01). Data were expressed as mean ± SD.

To verify the broad spectrum of the CRISPReader system in recognizing tumor cells, we transiently transferred the plasmid expressing the system into a series of gastric cancer cells and corresponding normal cells. The luciferase test results suggest that the CRISPReader can significantly activate the expression of luciferase in BGC-823, RPMI 1640, and HGC-27 gastric cancer cell lines that highly express Ets-1, while it cannot express luciferase in normal gastric epithelial cells GES-1 (Figure 3). The expression difference of the reporter gene suggests that the CRISPReader system can effectively distinguish gastric tumor cells from normal gastric cells, indicating that the Ets-1-sensing CRISPReader system has potential broad-spectrum anti-tumor capabilities.

### Inducing Cancer Cell Apoptosis

**FIGURE 4:**
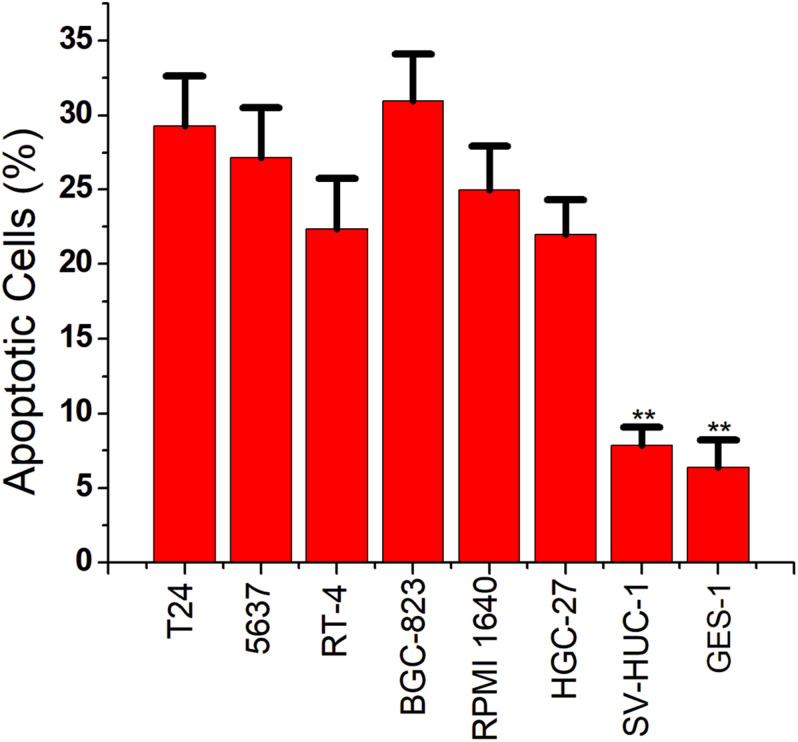
Specifically induced cancer cell apoptosis. Cell apoptosis activity was determined using the flow cytometry assay (**p < 0.01). Data were expressed as mean ± SD.

We then replaced the luciferase gene with the caspase-3 gene, which can induce apoptosis. The CRISPReader expression plasmid was transfected into bladder cancer and gastric cancer cell lines and the corresponding normal cell lines, respectively. The results of flow cytometry indicated that the apoptosis rate of tumor cell lines was significantly higher than that of normal cell lines (Figure 4), indicating that the Ets-1-sensitive CRISPReader system can effectively induce the apoptosis of different tumor cells and has broad-spectrum anti-tumor activity.

#### Inhibiting Cancer Cell Proliferation

**FIGURE 5:**
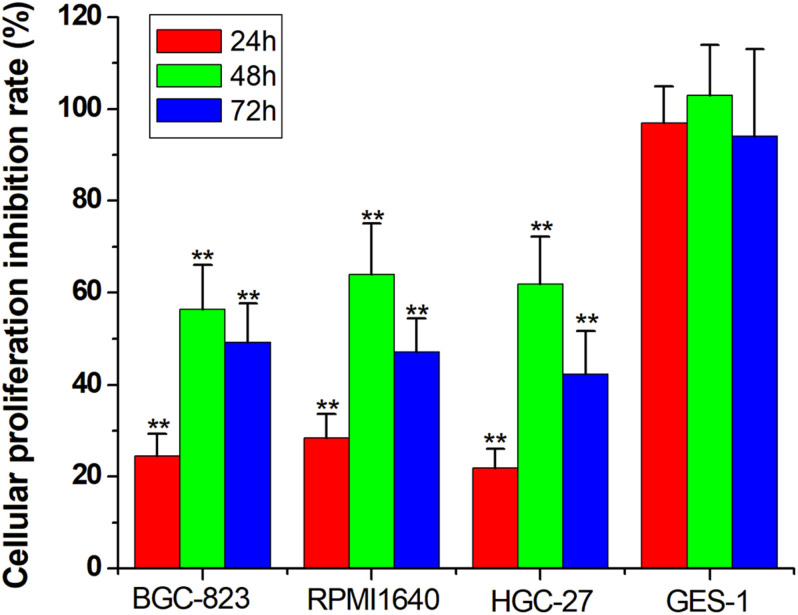
Specifically inhibited cancer cell proliferation. Cell proliferation rate was determined using the CCK-8 assay (**p < 0.01). Data were expressed as mean ± SD.

For the final step, we replaced the luciferase gene with the PTEN gene, which can inhibit cell proliferation. The CRISPReader expression plasmid was transfected into bladder cancer and gastric cancer cell lines and the corresponding normal cell lines, respectively. The results of the CCK-8 assay indicate that the proliferation rate of tumor cell lines was significantly lower than that of normal cell lines (Figure 5), indicating that the Ets-1-sensitive CRISPReader system can effectively inhibit the proliferation of different tumor cells and has broad-spectrum anti-tumor activity.

## Discussion

The application of the CRISPR system to the basic and therapeutic research of tumors is a major trend in current academic circles. An effective anti-cancer strategy is to use the CRISPR system to target fusion genes to exert anti-cancer effects (Zhan T. et al., 2019). However, the CRISPR system can not only regulate its downstream target genes but also sense a series of upstream signals, thus playing the role of the signal transmitter (Liu et al., 2018). It has obvious advantages when applied to tumor biotherapy, which can effectively identify and kill cancer cells. The biggest problem is how to choose the carcinogenic signal to be sensed. Ets-1 is a transcription factor that is generally highly expressed in tumors, so it is an ideal signal molecule for the CRISPR system.

In this study, we successfully designed a CRISPReader system that senses the Ets-1 transcription factor. It can effectively identify a variety of tumor cell lines including bladder cancer and gastric cancer, and specifically induce apoptosis and inhibit the proliferation of cancer cells. This study fully confirmed the effectiveness of Ets-1 as a broad-spectrum tumor recognition signal and provided a new anti-cancer tool based on the CRISPR system.

The limitation of this study is that only in vitro cell experiments were performed, and animal experiments will be needed in the future to further prove the anti-cancer effect of this tool.

## Data Availability Statement

The original contributions presented in the study are included in the article/supplementary material, further inquiries can be directed to the corresponding author.

## Author Contributions

KY provided the idea and designed the study details. All authors finished the experiments and wrote the manuscript, contributed to the article, and approved the submitted version.

## Conflict of Interest

The authors declare that the research was conducted in the absence of any commercial or financial relationships that could be construed as a potential conflict of interest.

## Footnotes

1 https://imagej.nih.gov/ij/
